# Association between AHR in EGCs and IBS-D patients: the indole pathway of tryptophan metabolism

**DOI:** 10.3389/fnut.2025.1566595

**Published:** 2025-03-28

**Authors:** Lianli Wang, Yue Zhang, Yan Ran, Laifu Li, Lin Mei, Fangchen Ye, Yating Sun, Ting Wang, Xiaojing Quan, Haitao Shi, Fei Dai

**Affiliations:** ^1^Division of Gastroenterology, The Second Affiliated Hospital of Xi’an Jiaotong University, Xi’an, China; ^2^Division of Gastroenterology, Honghui Hospital, Xi’an Jiaotong University College of Medicine, Xi’an, China

**Keywords:** tryptophan, aryl hydrocarbon receptor, inflammation, enteric glial cells, IBS-D

## Abstract

**Background:**

The pathophysiological mechanisms of irritable bowel syndrome (IBS) are intricate, and associated with tryptophan metabolites. This study was designed to investigate the relationship between indole metabolites in the feces and intestinal function in patients with IBS.

**Methods:**

In this study, 42 patients with diarrhea-predominant IBS (IBS-D) and 36 healthy controls were recruited. The symptom severity was evaluated using IBS-quality of life (IBS-QOL) and IBS symptom severity system (IBS-SSS). The levels of indole metabolite in fecal samples were determined by means of mass spectrometry. Colon mucosal tissues were collected during colonoscopy procedures. Immunohistochemistry or immunofluorescence techniques were employed to analyze the expressions of the aryl hydrocarbon receptor (AHR), cytochrome P450 1A1 (CYP1A1), glial fibrillary acidic protein (GFAP), S100 calcium-binding protein B (S100B), zonula occludens-1 (Zo-1), occludin, substance P (SP), nerve growth factor (NGF), NOD-like receptor family pyrin domain containing 3 (NLRP3), and nuclear factor kappa B (NF-κB) in the mucosal tissues.

**Results:**

Compared with healthy controls, the concentrations of the main indole metabolites (*p* = 0.020), and the expressions of CYP1A1 (*p* < 0.001), and Zo-1 (*p* = 0.017) were decreased in patients with IBS-D, but the expressions of S100B (*p* < 0.001), NF-κB (*p* = 0.006), and NRLP3 (*p* = 0.041) were increased. Immunofluorescence analysis demonstrated the co-expression of AHR with GFAP or S100B. Moreover, the ratio of S100B/AHR (*p* = 0.011) was higher in IBS-D patients than in health controls. This ratio was positively correlated with IBS-SSS score (*r* = 0.47, *p* = 0.006), as well as with the expression levels of NRLP3 (*r* = 0.505, *p* = 0.019), NF-κB (*r* = 0.548, *p* = 0.01), and SP (*r* = 0.832, *p* < 0.01).

**Conclusion:**

Patients with IBS-D exhibited low-grade inflammation in the colon mucosal tissues, compromised intestinal barrier function, and abnormal visceral sensation. This may be attributed to the decreased levels of tryptophan indole metabolites, the heightened activity of enteric glial cells (EGCs), and the inhibition of AHR/CPY1A1 signaling pathway.

## Graphical abstract

The pathophysiological mechanisms of irritable bowel syndrome (IBS) are related to the indole metabolic pathway of TRP. Our study is to emphasizes the association between AHR in EGC and intestinal function, and suggest that indole metabolites of TRP may have a protective effect on the gut by regulating AHR.
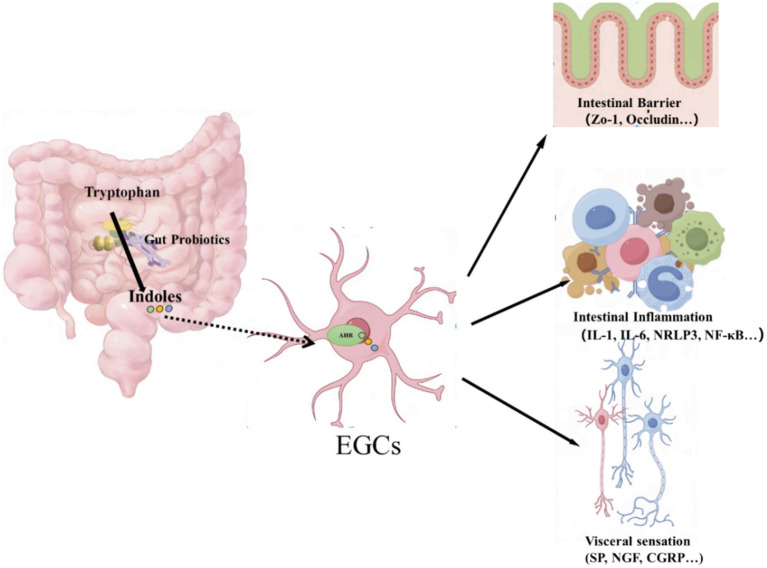

## 1 Introduction

Irritable bowel syndrome (IBS) is a functional gastrointestinal disorder characterized by chronic or recurrent abdominal symptoms. Multiple factors influence the complex pathogenesis of IBS (1). Low-grade inflammation or immune activation in the gut has been acknowledged as an important pathological feature of IBS (2, 3), particularly in development of visceral hypersensitivity and epithelial dysfunction. Additionally, the gut microbiota and microbiota-derived metabolites play crucial roles in regulating intestinal inflammation and function (4).

Accumulating evidence demonstrates that intestinal inflammation is closely associated with alterations in the metabolism of tryptophan (TRP), an essential amino acid that serves as the biochemical precursor of serotonin (5-hydroxytryptamine; 5-HT), kynurenine (KYN), and indoles (5). In animal models of inflammatory bowel disease and colitis, serotonin exerted both anti-inflammatory and pro-inflammatory effects within the intestinal microenvironment by binding to distinct 5-HT receptors (6). Quinolinic acid, kynurenic acid, and picolinic acid modulate gut immune function through the KYN pathway (7–9). The microbial metabolites of TRP, particularly indole metabolites, confer beneficial effects on the organism and are involved in the regulation of inflammation (10, 11). In recent years, only a limited number of studies have been carried out on the indole pathways of TRP in relation to IBS (12–14).

Indole metabolites function as potent modulators of immune function and regulate diverse physiological functions within the intestinal tract (10). The principal indole metabolites encompass indole, indole-3-acetic acid (IAA), indole-3-propionic acid (IPA), indoleacetic acid (IA), indole-3-lactic acid (ILA), and indole-3-aldehyde (IAld). Multiple studies have demonstrated that the IAA level was reduced in mice with dextran sulfate sodium (DSS)-induced colitis (15, 16). IA has the capacity to enhance intestinal epithelial barrier function and attenuate inflammatory responses by promoting goblet cell differentiation and mucus production (17). IAld can modulate interleukin-22 (IL-22)-dependent balanced mucosal responses through AHR, thereby conferring antifungal resistance and mucosal protection against inflammation (18). These indole metabolites serve as ligands of AHR, which have been shown to play a crucial role in regulating intestinal immunity and maintaining intestinal homeostasis (19). In addition to its role in immune and the epithelial cells, AHR plays a crucial role in the enteric nervous system (ENS) (20). EGCs, which are the main components of the ENS, are essential for the regulation of gastrointestinal functions (21). Our previous study (22) demonstrated that in IBS rats induced by acetic acid enema, there was activation of EGCs in colonic tissues, with the expression of GFAP and S100B increased. This was accompanied by an increased release of pro-inflammatory cytokines, such as tumor necrosis factor (TNF), interleukin-1β (IL-1β), and interleukin-6 (IL-6) from EGCs, as well as overexpression of the pain mediators, including SP, transient receptor potential vanilloid 1 (TRPV1), and CGRP. We hypothesize that indole metabolites might exert their effects in IBS via AHR-mediated regulation of the EGCs. In the present study, we investigated the alterations in fecal indole metabolites in patients with IBS-D and healthy controls, and explored the relationship between indole metabolites and EGCs-related gut functions.

## 2 Methods

### 2.1 Participants

Forty-two patients with IBS-D were diagnosed at the second affiliated hospital of Xi’an Jiaotong University in accordance with the Rome IV criteria and all were included in the subsequent analyses. Thirty-six healthy controls were recruited from individuals undergoing routine medical examinations with no manifestations of gastrointestinal (GI) symptoms. Exclusion criteria were the following: age <18 old, a history of abdominal surgery, abnormal thyroid function, abnormal liver function indices, kidney disease, active digestive tract infection, pregnancy, alcohol consumption, use of psychoactive medications or other drugs affecting gastrointestinal or psychoactive function within 7 days, or participation in other clinical trials within 3 months. Participants underwent colonoscopy after standard bowel preparation with polyethylene glycol, and mucosal pinch biopsies were obtained from the rectosigmoid junction. The study was approved by the Ethics Committee of the Second Affiliated Hospital of Xi’an Jiaotong University (No. 2022014) and was carried out in compliance with the Declaration of Helsinki. Written informed consent was obtained from all enrolled participants.

### 2.2 Sample collection

Fresh stool samples were collected using sterile plastic tubes from all participants between 7:00 am and 10:00 am. These samples were immediately transferred and stored at −80°C for subsequent analysis. All participants were instructed to maintain their normal dietary habits for at least 1 week prior to the stool sample collection and throughout the entire study period.

During colonoscopy, mucosal pinch biopsies were obtained from the rectosigmoid junction. The biopsies were immediately fixed in 10% formalin for a minimum of 72 h, embedded in paraffin, and sectioned to a thickness of 4 μm for immunohistochemistry analysis.

### 2.3 Questionnaire collections

The IBS Symptom Severity Scale (IBS-SSS) (23), is a 5-item self-reporting questionnaire specifically developed to assess the disease severity in individuals with irritable bowel syndrome. Each participant with IBS-D was provided this questionnaire for completion. The items within the questionnaire covered aspects such as abdominal pain, bloating, satisfaction with bowel habits, and overall interference with quality of life.

The IBS-Specific Quality of Life Questionnaire (IBS-QOL) (24) was employed to assess alterations in the quality of life of patients. This assessment covered eight distinct domains: dysphoria, interference with activity, body image, health worry, food avoidance, social reaction, sexual dysfunction, and relationships.

### 2.4 Feces tryptophan metabolomics

Fecal samples (25 mg) were weighed and then dissolved in 10 μL internal standard solution (the isotope standard, Trp-D5, was dissolved in methanol and diluted with ultrapure water to a concentration of 4,000 ng/mL), and 390 μL of the extraction solution (methanol: water = 4:1, v/v). The resulting mixture was homogenized and subsequently centrifuged at 14,000 g. The resulting mixture was homogenized and subsequently centrifuged at 14,000 g. The mixture was subjected to cryo-milling for 6 min (−10°C, 50 Hz), followed by cryo-sonication for 30 min (5°C, 40 kHz). It was left to stand at −20°C for 30 min. Then, it was centrifuged at 4°C and 13,000 RCF for 15 min. Three hundred microlitres of the supernatant was transferred and evaporated to dryness under a stream of nitrogen. Seventy-five microlitres of 1% acetonitrile-water solution (containing 0.1% formic acid) was added for re-dissolution. It was vortex-mixed for 30 s and underwent low-temperature ultrasonication for 15 min (5°C, 40 kHz). Finally, it was centrifuged at 4°C and 13,000 RCF for 15 min. The supernatant was then injected for high-performance liquid chromatography-tandem mass spectrometry (HPLC-MS/MS) analysis.

Briefly, the separation was performed on a ACQUITY UPLC^®^ HSST3 system (Agilent 1290 Infinity UHPLC) on a C-18 column (Waters, CSH C18 1.8 μm, 2.1 mm × 150 mm column) by gradient elution, with the column temperature maintained at 40°C. The injection volume was 2 μL. ABSCIEXQTRAP6500+ (AB SCIEX) was performed in the positive and negative ion mode. Default parameters in the AB Sciex quantitative software OS were utilized for the automatic identification and integration of individual ion fragments.

### 2.5 Mucosal tissue test

#### 2.5.1 Hematoxylin and eosin (H&E) staining

Tissue was fixed in 10% formalin, embedded in paraffin, and cut into 4-μm-thick sections. The sections were stained with hematoxylin-eosin (Servicebio, Wuhan, China) and imaged using an Olympus microscope (CX21, Olympus, Tokyo, Japan). H&E staining served to precisely examine the morphology of colonic mucosal tissue, and evaluate whether there was acute or chronic inflammation or a tumor.

#### 2.5.2 Immunohistochemistry (IHC) staining

Paraffin sections of the colon tissue were baked at 60°C for 1 h, followed by dewaxing in xylene (Sinopharm, Shanghai, China) and a series of gradient alcohol solutions (Sinopharm, Shanghai, China). Tissue antigen retrieval was carried out using a 10 mM sodium citrate solution (pH 6.0) (Servicebio, Wuhan, China) for 15 min at 100°C. After natural cooling to room temperature, the sections were treated with 3% hydrogen peroxidase (30 min, room temperature) (Sinopharm, Shanghai, China), and then incubated with 3% goat serum (Boster, Wuhan, China) (Servicebio, Wuhan, China) (60 min, room temperature). Subsequently, the sections were incubated with rabbit anti-human CYP1A1 (1:200, Affinity, Shanghai, China) overnight at 4°C. As a blank control, PBS was used instead of the primary antibody. After washing with PBS, the sections were incubated with a goat anti-rabbit secondary antibody (1:200, Affinity, Shanghai, China) for 45 min at 37°C. The specific immunoreactivity was detected using a DAB kit (Zenbio, Beijing, China). Images were acquired under a light microscope (CX21, Olympus, Tokyo, Japan). The expression intensity of CYP1A1 was quantified by mean density (MD) using ImageJ software (NIH, Bethesda, MD, United States).

#### 2.5.3 Immunofluorescence

Paraffin sections of the colon tissue were baked at 60°C for 1 h, followed by dewaxing in xylene (Sinopharm, shanghai, China) and a series of gradient alcohol solutions (Sinopharm, Shanghai, China). Tissue antigen retrieval was carried out using a 10 mM sodium citrate solution (pH 9.0) (Servicebio, Wuhan, China) for 15 min at 100°C. After the tissues were naturally cooled to room temperature, 0.3% Triton-100 (Servicebio, Wuhan, China) was added for permeabilization at room temperature for 30 min. Next, the sections were blocked with 3% goat serum (Boster, Wuhan, China). Multiple primary antibodies were then incubated with the sections overnight at 4°C. These included glial fibrillary acidic protein (GFAP) mouse-antibody (1:500, Servicebio, Wuhan, China), S100B rabbit-antibody (1:200, Affinity, Shanghai, China), SP rabbit-antibody (1:300, Affinity, Shanghai, China), NGF rabbit-antibody (1:200, Affinity, Shanghai, China), Zo-1 mouse-antibody (1:300, Proteintech, Wuhan, China), occludin rabbit-antibody (1:250, Proteintech, Wuhan, China), NRLP3 mouse-antibody (1:200, Proteintech, Wuhan, China), and NF-κB rabbit-antibody (1:200, Affinity, Shanghai, China). After washing with PBS, the sections were incubated with fluorescent secondary antibodies (fluorescent antibodies Cy3 conjugated goat anti-rabbit, and Alexa-488 conjugated goat anti-mouse, Servicebio, Wuhan, China). Finally, the tissues were counterstained with 4′,6-diamidino-2-phenylindole (DAPI, 2 μg/mL, Servicebio, Wuhan, China) and then observed under a fluorescence microscope (BX51, Olympus, Tokyo, Japan). Images were acquired and analyzed using the ZEN software (Carl Zeiss, Oberkochen, Germany) and Image J software (NIH, Bethesda, MD, United States). Data were presented as fluorescence intensity.

### 2.6 Statistical analysis

All statistical analyses were carried out using SPSS version 22 statistical software (SPSS Inc., Chicago, IL, United States). For all tests, two-sided *p*-values <0.05 were regarded as statistically significant. The normality of data distribution was assessed using the Kolmogorov–Smirnov test. Data were reported as mean ± standard deviation for parametric. Student’s *t*-test, *χ*^2^-test, or Fisher’s exact test were used between the two groups’ comparisons. Correlation coefficients were calculated according to either the Pearson or Spearman method.

## 3 Results

### 3.1 The characteristics of the subjects

Patients with IBS-D did not show differences from healthy controls in terms of age (34.10 ± 13.41 years vs. 37.00 ± 9.17 years, *p* = 0.61), gender (male: 72.5% vs. 57.9%, *p* = 0.20) or body mass index (BMI) (23.11 ± 3.29 vs. 22.86 ± 3.0, *p* = 0.78). Moreover, no significant abnormal findings were detected during endoscopy.

### 3.2 Fecal TRP metabolisms (indole pathway)

Tryptophan (TRP) indole metabolites are primarily produced via the interaction between the intestinal microbiota and dietary tryptophan. These metabolites include IPA, IA, IAA, IAId, and ILA (Table 1). Compared with the healthy controls, fecal tryptophan levels in patients with IBS-D were decreased, although the difference was not statistically significant (*p* = 0.196). Additionally, levels of indole metabolites in healthy controls were higher than those in IBS-D patients, such as IAA (*p* = 0.048), IPA (*p* = 0.040) and IAId (*p* = 0.012).

**Table 1:** The indole metabolites in fecal.

	Control health (ng/mg)	IBS-D group (ng/mg)	*p*
Indole propionic acid (IPA)	2.21 ± 2.28	1.21 ± 1.48	0.040
Indole acrylic acid (IA)	0.0082 ± 0.0042	0.0072 ± 0.0097	0.660
Indoleacetic acid (IAA)	2.66 ± 2.27	1.47 ± 1.88	0.048
Indole aldehyde (IAId)	1.53 ± 0.95	0.98 ± 0.80	0.012
Indole lactic acid (ILA)	1.92 ± 2.98	1.29 ± 1.84	0.813
The total above	8.48 ± 4.55	5.16 ± 3.85	0.002
Tryptophan	49.00 ± 23.01	42.91 ± 16.82	0.196

### 3.3 The expression of AHR and CYP1A1 in colon mucosa

**Figure 1:**
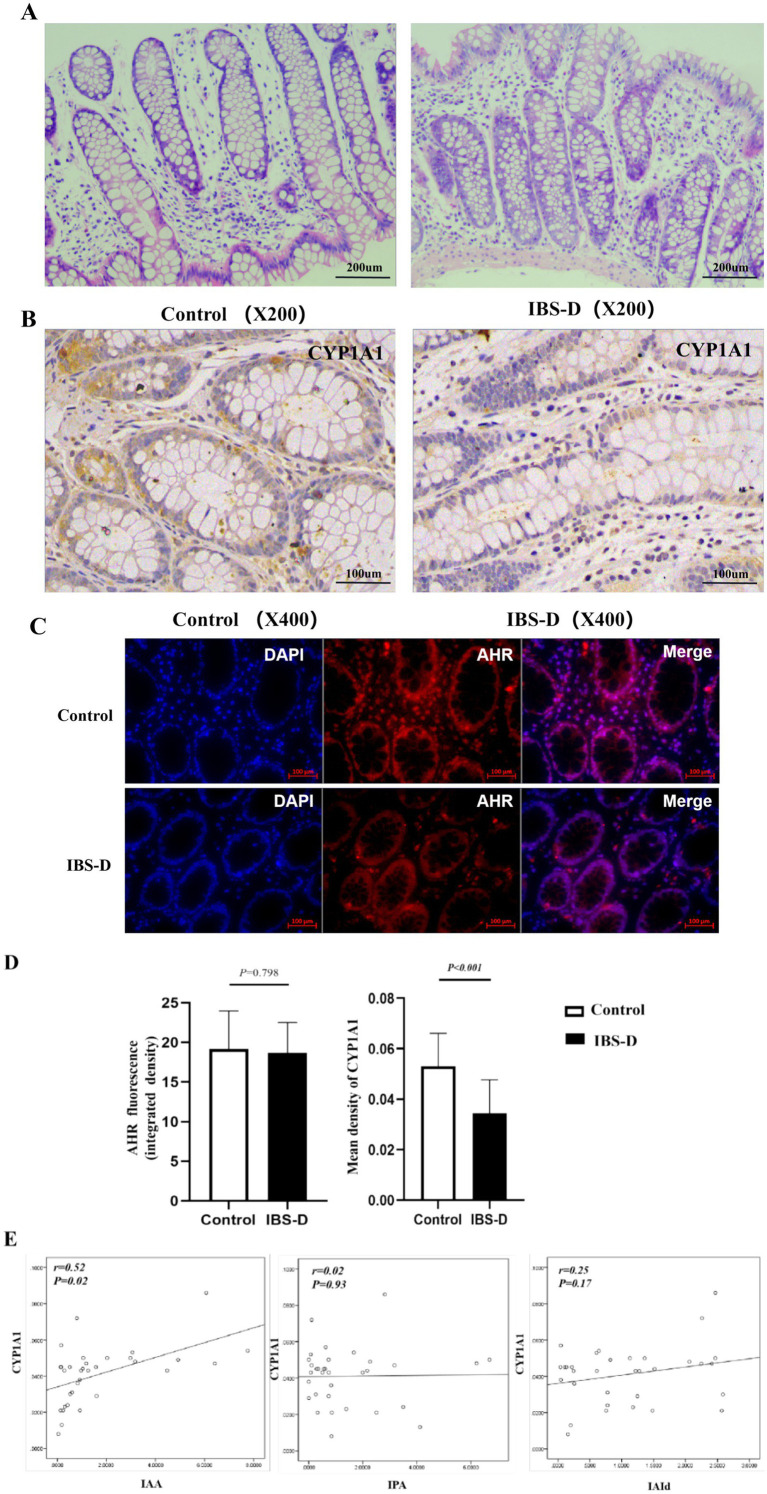
The expression of AHR and CYP1A1 in colon mucosa. **(A)** H&E of the colon mucosa of IBS-D patients and health controls. **(B–D)** The expression of AHR and CYP1A1 in the colon mucosa of IBS-D patients and health controls. **(E)** The relationship between the expression of CYP 1A1 and the main indole metabolites in fecal. IAA, indoleacetic acid; IPA, indole propionic acid; IAId, indole aldehyde.

H&E staining revealed that all colonic mucosal biopsies specimens exhibited normal and typical structures. There was no evidence of congestion, edema, ulcers, or evident infiltration of inflammatory cells (Figure 1A).

In the colonic mucosa, the level of the AHR fluorescence intensity in the colonic mucosa of IBS-D patients was decreased with no significantly different from that in healthy controls (18.66 ± 3.86 vs. 19.17 ± 4.82, *p* = 0.798) (Figures 1C,D). CYP1A1 is a well-recognized marker of AHR activation. IHC demonstrated that the CYP1A1 was positively stained brownish-yellow in mucosal biopsies and was expressed in the cytoplasm of intestinal epithelial cells, immune cells, and other cell types (Figure 1B). And the expressive level of CYP1A1 was significantly decreased in the IBS-D patients, compared to the healthy controls (0.034 ± 0.013 vs. 0.053 ± 0.013; *p* < 0.001) (Figures 1B,D). Interestingly, the expression of CYP1A1 was positively associated with the levels of fecal tryptophan (TRP) indole metabolites, particularly with IAA (*r* = 0.52, *p* = 0.02) (Figure 1E).

### 3.4 The co-expression of GFAP or S100B and AHR in the colon mucosa

**Figure 2:**
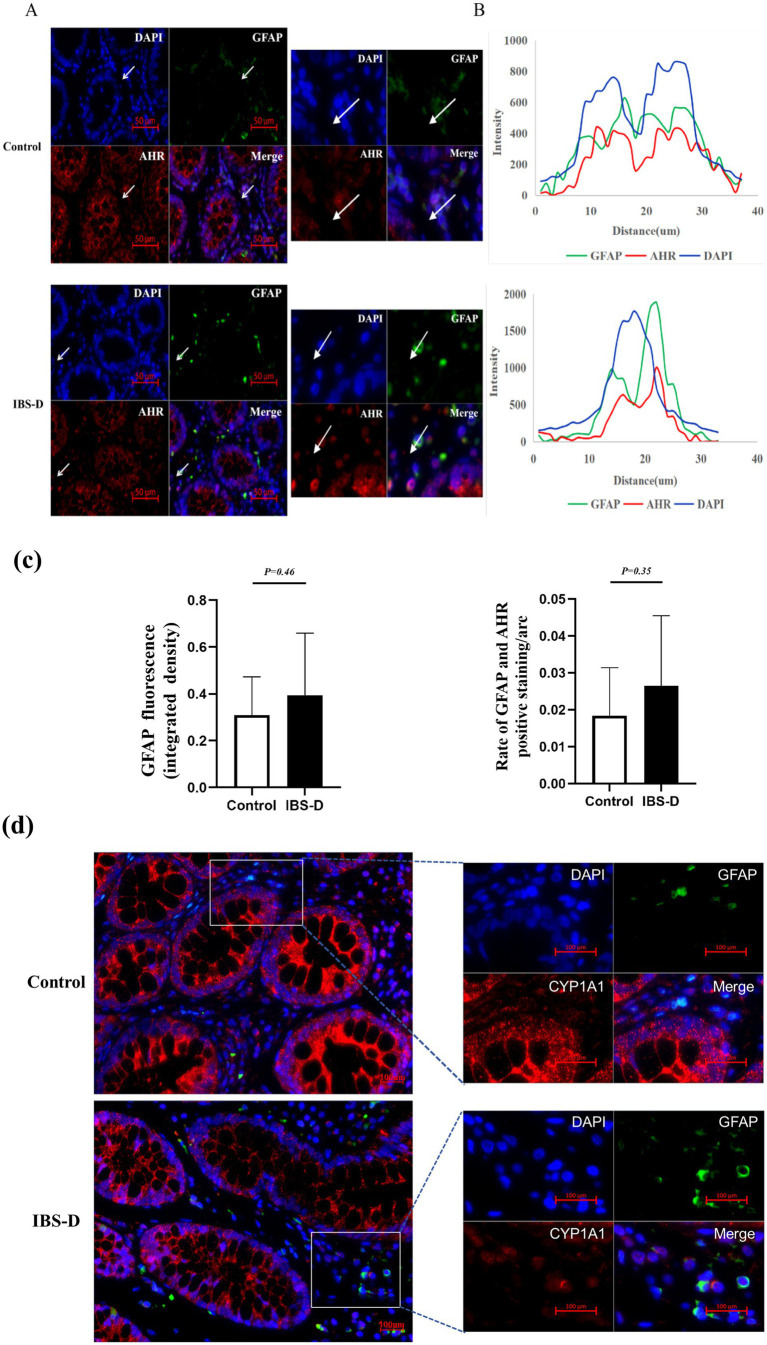
The co-expression of GFAP and AHR in the colon mucosa. **(A,B)** Sample images showing overlays of AHR (red) and GFAP (green) staining in the colon mucosa of IBS-D patients and health controls. **(C)** The expression of GFAP and the overlap of AHR and GFAP. **(D)** The CYP1A1 expression within EGCs.

**Figure 3:**
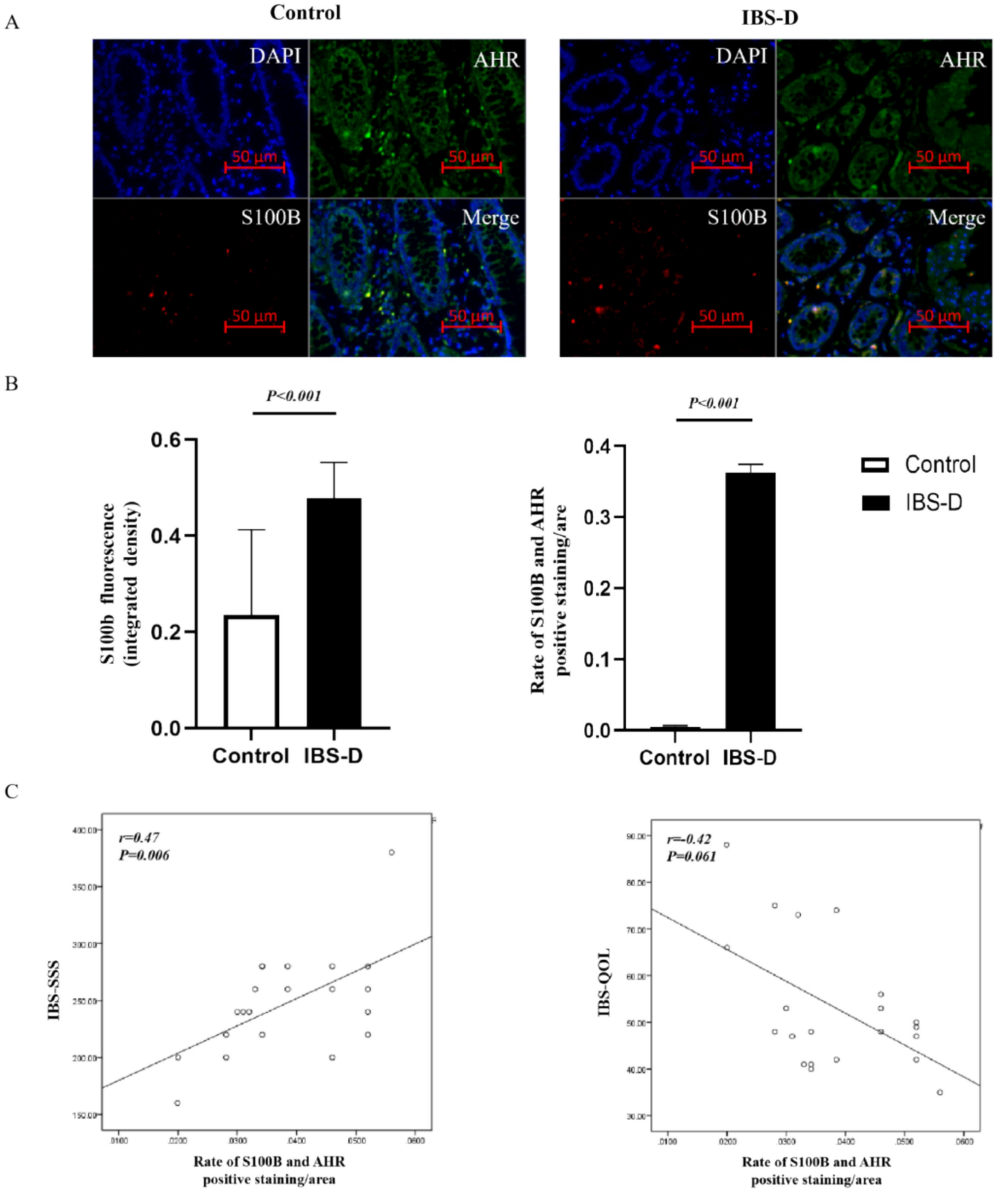
The co-expression of S100B and AHR in the colon mucosa. **(A,B)** The expression of AHR (green) and S100B (red) in the colon mucosa of IBS-D patients and health controls. **(C)** The relationship between the rate of S100B and AHR and the Symptom Score.

GFAP and S100B are specific markers for the maturation and activation of enteric glial cells (EGCs). The upregulation of their expression reflects the activated state and enhanced functionality of EGCs. Immunofluorescence indicated that GFAP and S100B were positively stained as green fluorescence or red fluorescence, and were expressed in the cells of the subepithelial lamina propria and peri glandular areas within the colon mucosa. Double-staining experiments verified that the expression of GFAP or S100B was colocalized with AHR or CYP1A1 in the colonic mucosa, indicating that AHR or CYP1A1 was expressed on EGCs (Figures 2, 3). Compared with the healthy controls, the expression level of GFAP (0.393 ± 0.266 vs. 0.308 ± 0.164, *p* = 0.455) and S100B (0.479 ± 0.074 vs. 0.235 ± 0.178, *p* < 0.001) in the colon mucosa of IBS-D patients was increased. And the ratio of S100B/AHR was significantly higher in IBS-D patients than in the healthy controls (0.363 ± 0.011 vs. 0.006 ± 0.002, *p* < 0.001). However, there was no significant difference in the rate of GFAP/AHR between IBS-D patients and healthy controls (0.0265 ± 0.019 vs.0.018 ± 0.014, *p* = 0.352). In IBS-D patients, a positive correlation was observed between IBS-SSS and the ratio of S100B/AHR (*R* = 0.470, *p* = 0.006). Meanwhile, the ratio of S100B/AHR showed a tendency towards a negative correlation with IBS-QOL (*r* = −0.425, *p* = 0.061), although the difference was not statistically significant.

### 3.5 The level of Zo-1 and occludin in the colon mucosa

**Figure 4:**
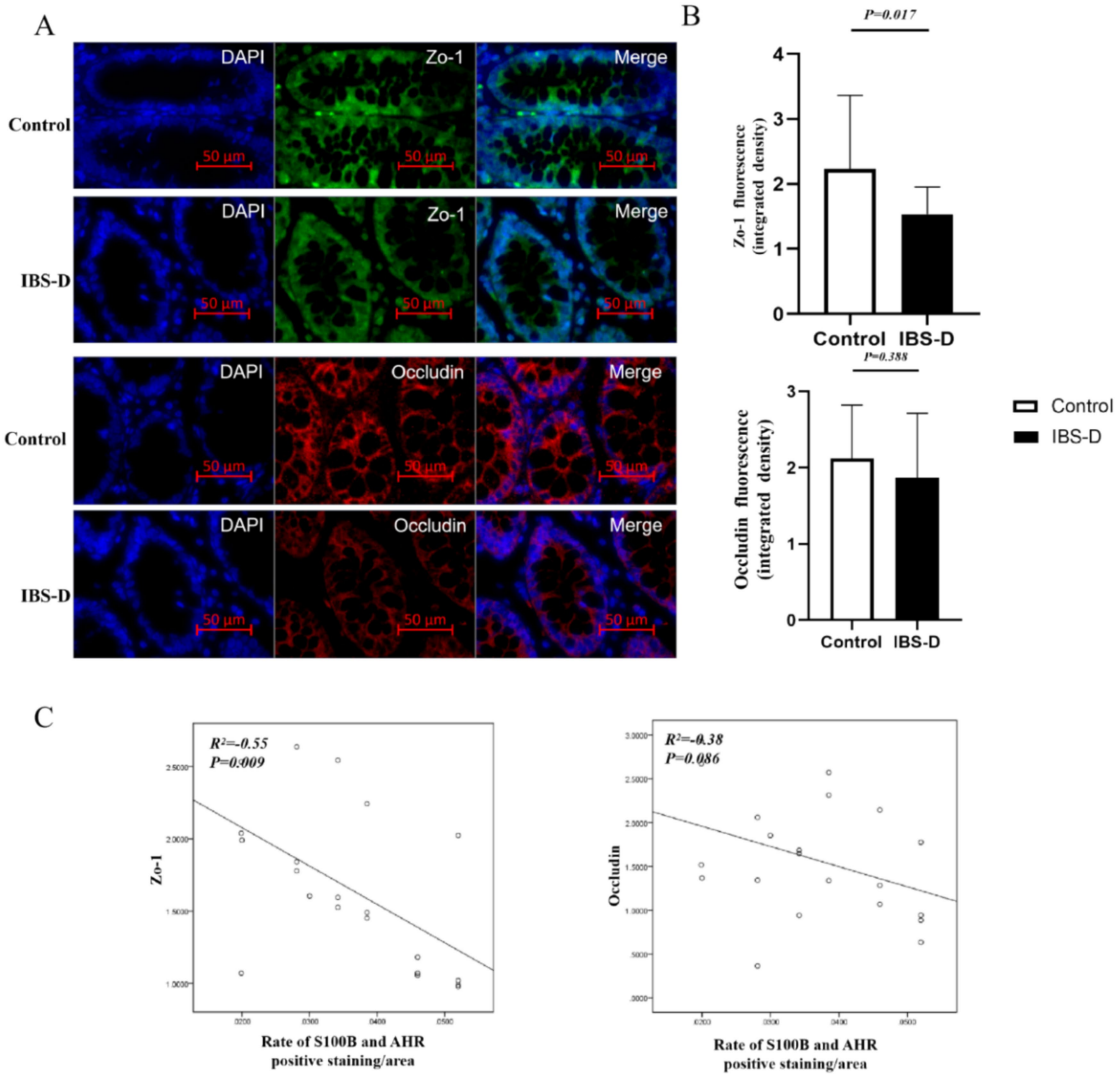
The level of Zo-1 and occludin in the colon mucosa. **(A,B)** The expression of Zo-1 and occludin. **(C)** The relationship between the rate of S100B and AHR and the expression of Zo-1 or occludin.

Zo-1 and occludin are critical core components of tight junctions, whose expression levels and spatial distribution directly reflect the structural and functional integrity of the intestinal epithelial barrier. Downregulation of their expression is closely associated with increased intestinal permeability and the progression of intestinal disorders. Compared with healthy controls, the expression levels of Zo-1 (2.234 ± 1.132 vs. 1.535 ± 0.421, *p* = 0.017) and occludin (2.120 ± 0.702 vs. 1.871 ± 0.843, *p* = 0.388) were decreased in IBS-D patients. And the expression levels of Zo-1 and occludin were negatively correlated with the ratio of S100B/AHR (*r* = −0.554, *p* = 0.009; *r* = −0.383, *p* = 0.086) (Figure 4).

### 3.6 The level of NGF and SP in the colon mucosa

**Figure 5:**
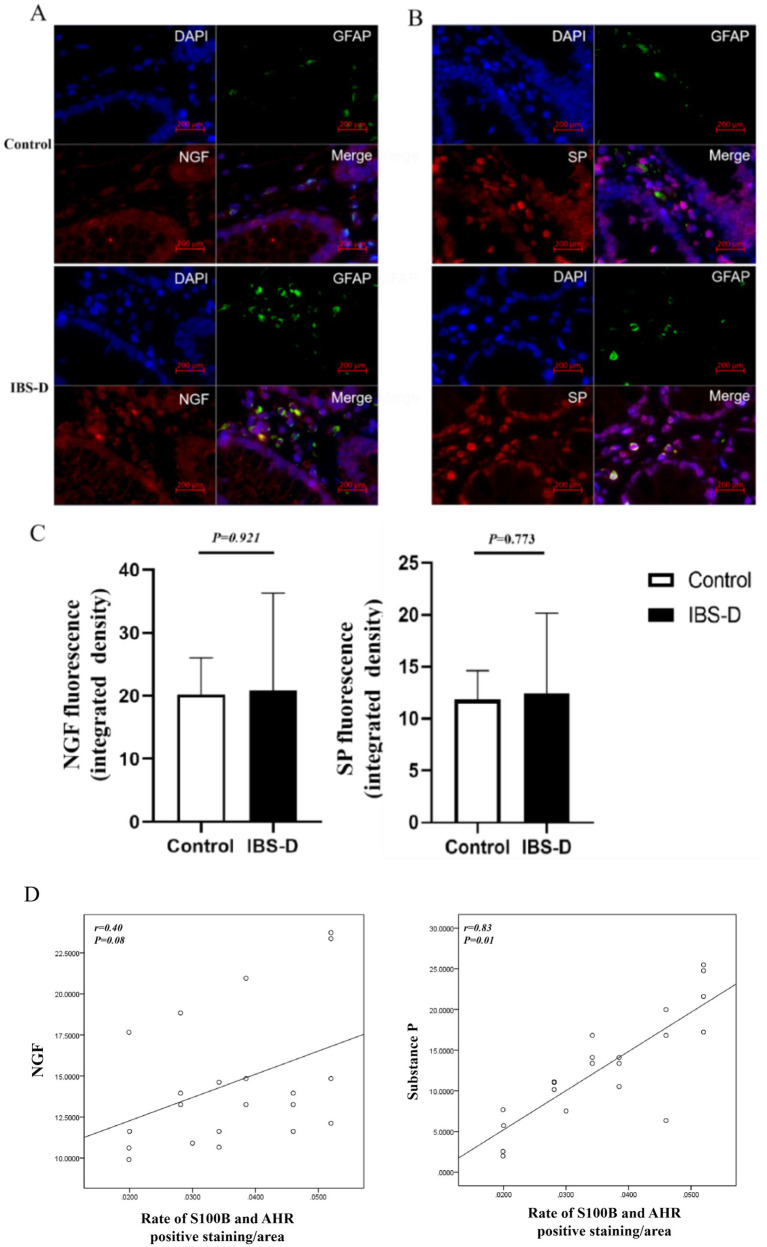
The level of SP and NGF in the colon mucosa. **(A–C)** The expression of SP and NGF. **(D)** The relationship between the rate of S100B and AHR and the expression of SP or NGF.

SP and NGF are pivotal mediators in pain signaling and modulation, playing critical roles in the initiation, progression, and persistence of pain. Elevated levels of SP and NGF are strongly associated with the severity of abdominal pain symptoms, particularly in visceral hypersensitivity disorders such as irritable bowel syndrome (IBS). There was no significant difference of the expression levels of NGF (20.190 ± 5.848 vs. 20.859 ± 15.423, *p* = 0.921) and SP (11.822 ± 2.816 vs. 12.424 ± 7.73, *p* = 0.773) between the healthy controls and IBS-D patients. However, the expression levels of NGF and SP were positively correlated with the ratio of S100B/AHR (*r* = 0.395, *p* = 0.076; *r* = 0.832, *p* = 0.009). Double immunofluorescence staining demonstrated that EGCs in colon tissue expressed SP and NGF (Figure 5).

### 3.7 The level of NF-κB and NRLP3 in the colon mucosa

**Figure 6:**
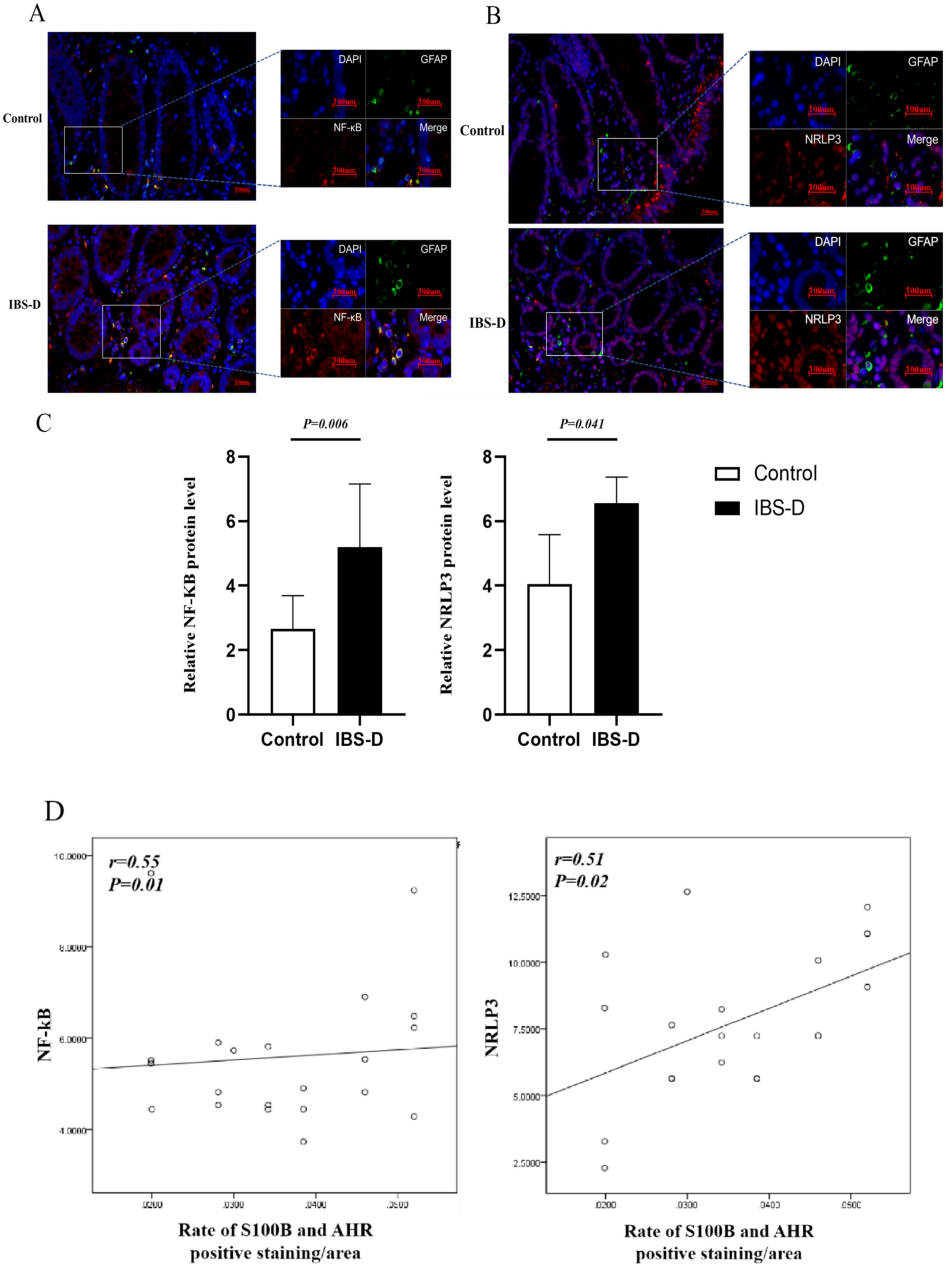
The level of NF-κB and NRLP3 in the colon mucosa. **(A–C)** The expression of NF-κB and NRLP3. **(D)** The relationship between the rate of S100B and AHR and the expression of NF-κB or NRLP3.

Nuclear factor kappa B (NF-κB) is a critical transcription factor regulating inflammation, immune responses, and cell survival. NLRP3, a core component of the inflammasome and a key member of the NOD-like receptor (NLR) family, mediates innate immune responses. Compared to the healthy controls, the expression levels of NF-κB (5.196 ± 1.960 vs. 2.663 ± 1.026, *p* = 0.006) and NRLP3 (6.557 ± 3.262 vs. 4.040 ± 1.545, *p* = 0.041) were elevated in IBS-D patients. Additionally, the expression levels of NF-κB and NRLP3 were positively associated with the ratio of S100B/AHR (*r* = 0.548, *p* = 0.010; *r* = 0.505, *p* = 0.019). Furthermore, double immunofluorescence staining showed that NF-κB and NRLP3 were co-localized with GFAP-immunopositive EGCs in colon tissue (Figure 6).

## 4 Discussion

This study preliminarily investigated the potential role of AHR and EGCs in the pathogenesis of IBS-D. The expression level of CYP1A1 in the colonic mucosa was significantly reduced in IBS-D patients and was correlated with fecal indole metabolites. In contrast, there was no significant difference in AHR expression between IBS-D patients and healthy controls. Additionally, the co-expression of S100B and AHR in IBS-D patients differed from that in controls and was associated with IBS-SSS, intestinal barrier function, visceral hypersensitivity, and intestinal inflammation. To the best of our knowledge, few studies have explored the relationship between the tryptophan (TRP) indole pathway and gut function in IBS-D patients.

Microbial TRP catabolism in the colon generates indole metabolites (25), such as IAA, IAId, IA, IAld, and tryptamine. These metabolites serve as potent endogenous ligands of AHR, regulating the expression of CYP1A1 and the transcription of multiple genes, including IL-1, IL-4, IL-6, IL-17, IL-10, IL-23, IL-27, and IFN-γ (26). Previous studies (14, 27, 28) have focused on the KYN and 5-HT metabolic pathways of TRP in the context of, with relatively less attentions directed towards the indole derivative metabolic pathway. In our study, the microbial-derived sources of indoles, especially IAA, ILA, and IPA, were significantly reduced in patients with IBS-D, as well as the expression level of CYP1A1. And a positive correlation was observed between CYP1A1 and indole metabolites. Given that CYP1A1 is a marker of AHR activation, the findings presented herein indicate that the function of AHR is inhibited in the intestinal mucosa of IBS-D patients.

The luminal contents of the gastrointestinal tract serve as a rich source of TRP and support microbial metabolic activity (29). Modulating the composition of intestinal microflora and its metabolites can enhance intestinal function. A study has shown that in bifidobacterial-dominated breastfed infants, increased production of ILA could protect gut epithelial cells by activating the AHR and nuclear factor erythroid 2-related factor 2 (Nrf2) pathways (30). *Bacteroides ovatus*-produced IAA stimulates the production of IL-22 by immune cells, exerting beneficial effects in colitis (31). Administration of nicotine has been associated with increased indole levels in fecal samples and has been shown to alleviate DSS-induced colitis in mice (32). Fu brick tea brownin-induced increases in IAld and IAA can activate aromatic AHR and boost the production of IL-22, thereby facilitating the repair of the intestinal barrier (33). Consistent with our findings, patients with IBS-D exhibited decreased levels of indole metabolites in their feces. Concurrently, in the colonic mucosa, the expression of CYP1A1 decreased, while the expression of NF-κB, NRLP3, NGF, and SP increased, and the expression of Zo-1 and occludin decreased. Furthermore, the indole metabolites, acting as ligands for AHR, regulate gut function by influencing both epithelial and immune cells. EGCs, as neuroimmune cells, also play a crucial role in the regulation of the gastrointestinal tract (21). Our study is the first to report the co-expression of AHR and GFAP in colonic mucosal specimens of patients with IBS-D. The role of AHR in EGCs is associated with intestinal inflammation, intestinal barrier function, and pain-related molecules intestinal barrier function.

EGCs are an important component of ENS and are distributed across all layers of the gastrointestinal tract. They play a crucial role in maintaining the homeostasis of the internal environment by regulating intestinal secretion, motility, and the integrity of epithelial barrier (21). GFAP and S100B are well-known markers for glial cell markers (34). GFAP, a specific marker indicating the maturation of EGCs, is regulated by factors, such as cell differentiation, inflammation, and injury (35). During gastrointestinal tract inflammation, S100B, a Ca2^+^-binding protein, can upregulate the expression of pro-inflammatory cytokines, chemokines and other inflammatory mediators through the mitogen-activated protein kinase (MAPK) and NF-κB signaling pathways, thereby promoting the progression of inflammation (36). In our study, the expression levels of GFAP and S100B were increased, which was accompanied by an increase in the expression of NF-κB and NRLP3. Our previous research also revealed that in patients with functional dyspepsia (FD), the increased expression of GFAP was positively correlated with epigastric pain symptoms (37). And compared to control rats and enteric glial cells, the expression levels of GFAP, S100B, SP, CGRP, TRPV1, TNF, IL-1β, and IL-6, were significantly higher in the colonic tissues of irritable bowel syndrome (IBS) rats and lipopolysaccharide (LPS)-treated EGCs (22). Collectively, these studies suggest that abnormal EGCs function has an impact on gastrointestinal function. However, the precise underlying mechanisms by which EGCs exert their effects remain incompletely.

Recently, the role of AHR in EGCs has not been fully investigated. AHR, a transcription factor, translocates into the nucleus binding to its ligands. Subsequently, it forms a heterodimer with the aryl hydrocarbon receptor nuclear translocator (ARNT). This complex then induces the expression of downstream genes, such as CYP1A1, and the regulates processes including cell proliferation, apoptosis, and inhibition of immune response (38). EGCs share similarities with astrocytes. The microbial TRP metabolites, such as 3-indoxyl sulfate, IPA and IAId, can mitigate central nervous system (CNS) inflammation and neurodegeneration inflammation by activating AHR signaling pathway in astrocytes (39). In AHR-deficient astrocytes, the binding of NF-κB to the Ccl2, Csf2 and Nos2 promoters was enhanced. This indicates that AHR negatively regulates the activation of NF-κB in astrocytes (40). Notably, there has been no previously published research exploring the relationship between AHR and EGCs in the context of intestinal function. Our study reveals new insights into the association between AHR expression in EGCs and key pathological features of intestinal disorders, including inflammation, barrier dysfunction, and visceral hypersensitivity.

Our study has some limitations: first, we did not measure serums levels of indoles or characterize the microbiota of the study participants, which would add important information to this study. Second, food consumption was not recorded in this study. Diet is a key source of TRP, and may affect TRP metabolic levels.

## 5 Conclusion

This study offers new evidence regarding the role of the indole TRP metabolic pathway in IBS-D and underscores the potential link between AHR activation in EGCs and intestinal function. These findings imply that TRP indole metabolites might exert a protective effect on the gut by modulating AHR. Nevertheless, the underlying mechanisms remain to be elucidated, and these findings pave the way for new directions in future research.

## Data Availability

The original contributions presented in the study are included in the article/supplementary material, further inquiries can be directed to the corresponding author.
